# Gammaretroviruses tether to mitotic chromatin by directly binding nucleosomal histone proteins

**DOI:** 10.15698/mic2018.08.643

**Published:** 2018-07-24

**Authors:** Madushi Wanaguru, Kate N. Bishop

**Affiliations:** 1 Retroviral Replication Laboratory, The Francis Crick Institute, London, NW1 1AT, United Kingdom.

**Keywords:** retrovirus, murine leukemia virus, Gag, p12, nucleosomes, integration, chromatin-targeting

## Abstract

The gammaretroviral gag cleavage product, p12, is essential for replication at both early and late stages of the virus life cycle. During the early stage of infection, the viral core is released into the cytoplasm, the viral RNA genome is reversed transcribed to cDNA and this viral DNA is then integrated into the host cell chromatin to form a provirus. The p12 protein has N- and C-terminal domains (NTD and CTD) that are required for steps leading up to integration, but the molecular details of their functions remain poorly characterised. Using the prototypic gammaretrovirus, murine leukemia virus (MLV) as a model, we recently showed that the NTD of p12 directly binds to and stabilises the capsid (CA) lattice of the viral core. Alterations to the CTD of MLV p12 prevented the viral pre-integration complex (PIC) tethering to host chromatin in mitosis, and this could be partially rescued by addition of a heterologous chromatin binding motif. In this study we demonstrated that the CTD of p12 directly binds to nucleosomal histone proteins, targeting not only p12 but also CA to mitotic chromatin. Additionally, cell-cycle-dependent phosphorylation of p12 appeared to increase the affinity of p12 for chromatin in mitosis relative to interphase. Thus, we have revealed how p12 can link the CA-containing PIC to mitotic chromatin, ready for integration. Importantly, we observed that direct binding to nucleosomes is a conserved feature of p12 orthologs across the gammaretrovirus genus and that the nucleosomal docking site is potentially shared with that of spumaretroviral Gag proteins.

## INTRODUCTION

**Figure 1:**
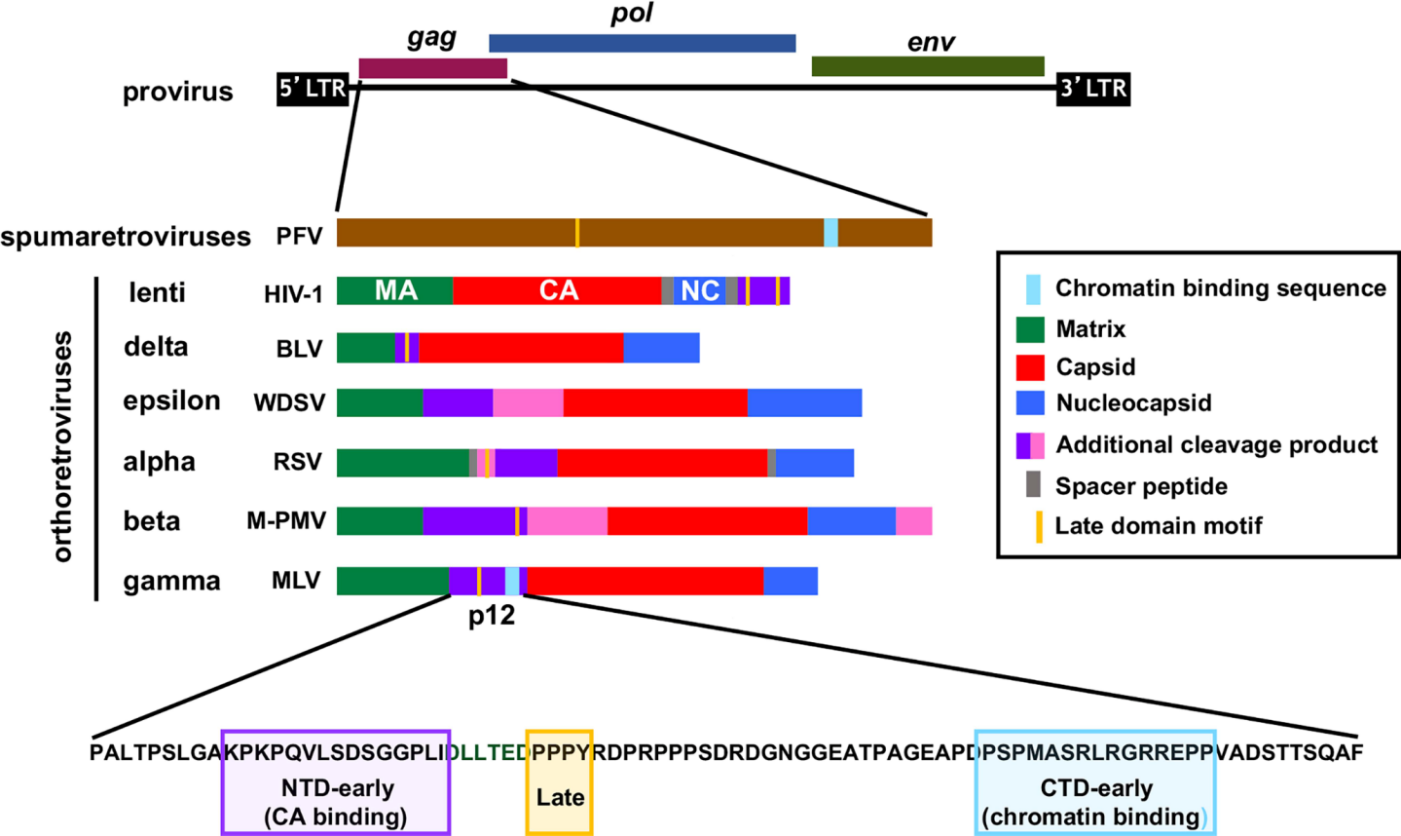
FIGURE 1: Schematic representation of retroviral Gag proteins. All retroviruses encode three main genes: gag, pol and env. The gag gene codes for the structural proteins of the virus. It is translated into a polyprotein that is then cleaved during viral maturation in orthoretroviruses into individual proteins as indicated in the key: matrix (MA, green); capsid (CA, red); nucleocapsid (NC, blue) and additional small proteins (purple and pink). The Gag protein of spumaretroviruses is not cleaved, shown in brown. The position of proline-rich late domain motifs which recruit the ESCRT pathway to facilitate viral release in late replication are indicated in yellow. The identified chromatin binding motifs are shown in light blue. The p12 amino acid sequence from the gammaretrovirus Mo-MLV is given, highlighting the motif in the NTD responsible for CA binding (in purple) and the CTD chromatin binding motif (in light blue). Abbreviations: PFV, prototypic foamy virus; HIV, human inmmunodeficiency virus; BLV, bovine leukemia virus; WDSV, walleye dermal sarcoma virus; RSV, Rous sarcoma virus; M-PMV, Mason Pfizer Monkey virus; MLV, murine leukemia virus.

The retroviral *gag* gene codes for all the structural components of the virus, except the envelope glycoproteins. During virion maturation, the Gag polyproteins of orthoretroviruses, but not spumaretroviruses, are proteolytically cleaved to generate a number of separate proteins including the three main structural proteins, matrix (MA), CA, and nucleocapsid (NC) (Figure 1). In addition, many orthoretroviruses also produce other Gag cleavage products which perform ill-defined functions (purple and pink coloured proteins in Figure 1). These proteins frequently carry proline-rich late (L)-domains that facilitate virus budding from the plasma membrane by recruiting cellular proteins of the endosomal sorting complexes required for transport (ESCRT) pathway to Gag assemblies. Additionally, the lentiviral accessory proteins Vpr and Vpx are packaged into viral particles by binding to the p6 region of lentiviral Gag proteins during assembly. On the other hand, the p12 proteins of gammaretroviruses are also essential for events in early replication, including nuclear/chromatin targeting.

Integration of the viral genome in to host chromatin is a hallmark of retroviral replication. Gaining entry into the nucleus of target cells is therefore a fundamental step of the retroviral life cycle. Interestingly, whereas retroviral integrase (IN) enzymes catalyse the process of integration itself and, by binding to cellular co-factors, direct specific integration site selection to particular features of chromatin, emerging evidence suggest that the initial ‘global’ nuclear/chromatin targeting of retroviral PICs may be mediated by Gag proteins. HIV-1, which replicates efficiently in both cycling and non-cycling cells, actively traverses the nuclear envelope and locates active chromatin with the aid of CA-binding host proteins, including nucleoporin 153 (Nup153) and cleavage and polyadenylation specificity factor 6 (CPSF6). In the case of spumaretroviruses, which have a limited ability to cross the nuclear membranes, tethering of the PIC to host chromatin occurs in mitosis and also seems to be directed by their largely un-cleaved Gag protein. In particular, chromatin tethering of the spumaretrovirus prototypic foamy virus (PFV) is known to be mediated by the direct binding of Gag to nucleosomal histones H2A and H2B via a short, conserved sequence motif called the chromatin binding sequence (CBS) (Figure 1). Like spumaretroviruses, gammaretroviruses are also dependent on the disassembly of the nuclear envelope in mitosis for gaining access to host chromatin. Viral genome integration is thought to take place following de-condensation of chromatin. However, it is likely that the gammaretroviral PIC first interacts with mitotic chromatin in order to be retained in the nucleus, and recent observations suggest that their Gag cleavage product, p12, may play an important role. Alanine substitutions in the CTD of Mo-MLV p12 prevent chromatin docking of PICs and decrease infectivity by more than a 100-fold. Compellingly, both chromatin tethering and infectivity of these mutants can be rescued, albeit partially, by inserting heterologous CBSs into p12.

The aim of this study was to elucidate the molecular details of p12-chromatin interactions using a combination of virological and biochemical approaches. As CA and p12 are known to be components of the MLV PIC, we hypothesised that p12 tethers the viral PIC to chromatin by binding CA and chromatin simultaneously, via the NTD and CTD regions respectively. Indeed, by immuno-staining Mo-MLV infected cells, we observed that p12 and CA co-localise on mitotic chromatin in a p12 CTD-dependent manner. Importantly, the association of p12/CA with mitotic chromatin was not significantly affected by a mutation in IN which prevents its interaction with chromatin-associated bromodomain and extraterminal domain (BET) proteins. This suggests that mitotic chromatin tethering of the CA-containing MLV PIC is primarily driven by p12 and not IN.

In cells infected with Mo-MLV, p12 has only been observed to associate with chromatin in mitosis. Whether this observation reflects a preferential affinity of p12 for mitotic chromatin above interphase chromatin, the accessibility of chromatin during infection or the involvement of other viral factors is not known. To investigate the cell-cycle-dependency of p12-chromatin interactions directly, we compared the chromatin tethering of a recombinant GST- tagged Mo-MLV p12 fusion protein in interphase and mitotic cells. In microscopy assays, GST-p12 showed a chromatin localisation in mitotic cells but appeared to have a predominantly cytosolic distribution in interphase cells. This apparent preference of GST-p12 for mitotic chromatin was corroborated by its significantly higher precipitation of histone proteins from mitotic cell lysates in pull-down assays. Intriguingly, we observed that the cell-cycle-dependent chromatin binding of GST-p12 correlated with its state of phosphorylation. Like viral Mo-MLV p12, GST-p12 was also phosphorylated mainly at serine-61 in the CTD. In our assays, GST-p12 was significantly more phosphorylated in mitosis compared to interphase, suggesting that phosphorylation of p12 may be increasing its affinity for chromatin. Indeed, kinase-inhibiting drugs and phosphoablatory mutations which decreased GST-p12 phosphorylation also reduced its binding to chromatin.

To identify the chromatin target of MLV p12 we used a stable isotope labelling in cell culture-mass spectrometry (SILAC-MS) approach to characterise the interactions of GST-tagged Mo-MLV p12. Our results suggest that the chromatin interactome of GST-p12 comprises of ~70 proteins. Of these, nucleosomal histone proteins showed the highest normalised abundance in pull-down elutions, indicating that p12 may be binding directly to nucleosomes. This was confirmed in BLI (bio-layer interferometry) assays by probing the binding of MLV p12 CTD peptides to recombinant poly-nucleosomal arrays. Importantly, direct binding to nucleosomes appears to be a conserved feature of p12 orthologs across the retrovirus genus. Feline leukemia virus and gibbon ape leukemia virus p12 proteins precipitated nucleosomal histone proteins in pull-down assays when expressed as GST-fusion proteins and showed binding to recombinant nucleosomes in BLI assays.

Like gammaretroviral p12, the Gag protein of the spumaretrovirus PFV also binds directly to nucleosomes. Indeed, our SILAC-MS analysis revealed a very significant overlap of ~90% between the chromatin interactomes of recombinant p12 and the PFV CBS. Interestingly, in BLI assays, p12 CTD peptides were able to inhibit the binding of the PFV CBS to nucleosomes, and *vice versa,* suggesting that they may share a binding interface. PFV is known to bind nucleosomes at an acidic patch at the H2A-H2B heterodimeric interface, which is also targeted by the latency-associated nuclear antigen (LANA) protein of Kaposi’s sarcoma associated herpes virus (KSHV). Both PFV Gag and KSHV LANA carry a conserved arginine residue in their CBSs that forms critical contacts with the carboxylate groups in the nucleosomal acidic pocket. Compellingly, the CTD regions of gammaretroviral p12 proteins also contain a number of conserved arginine residues which are essential for replication and chromatin tethering. The mechanism of nucleosome binding is therefore likely to be similar between these different viral proteins.

**Figure 2:**
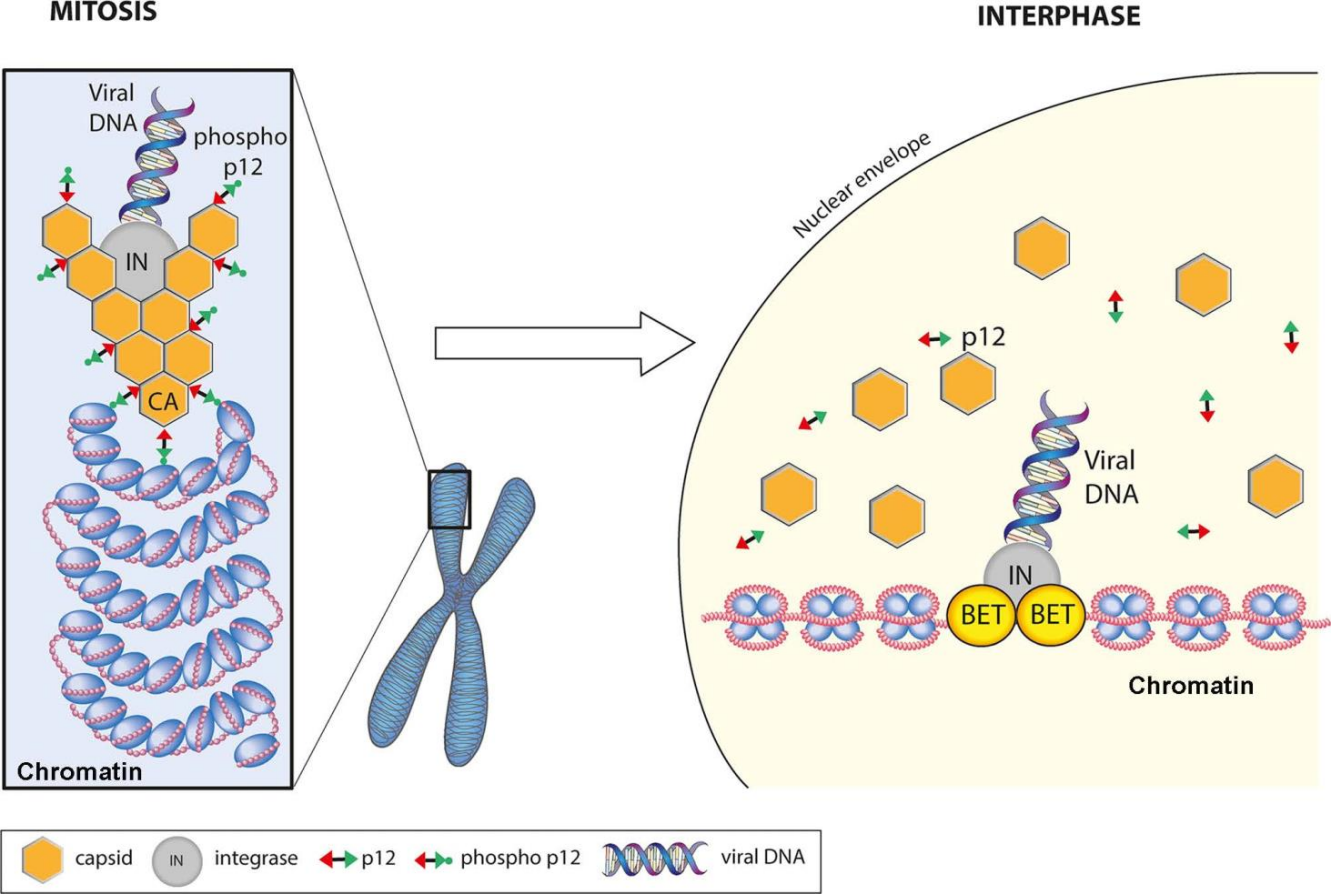
FIGURE 2: A model for gammaretroviral p12 function in the early stages of infection. In the mature virion, the NTD of p12 directly binds to and stabilises the hexameric CA lattice that surrounds the viral core. The gammaretroviral PIC, minimally carrying p12, CA, IN and the viral cDNA must wait until mitosis, when the nuclear envelope disassembles, to gain access to host chromatin. The PIC is then tethered to nucleosomes by CA-bound, phosphorylated p12. Exit from mitosis triggers the de-phosphorylation of p12 and the dissociation of p12 and CA from chromatin. This then reveals the intasome, consisting of viral cDNA and IN. IN can then bind to chromatin-associated BET proteins and direct the viral cDNA to gene promoter regions where it then catalyses integration of the viral cDNA into host chromatin to form a provirus.

Putting all the data together, we propose the following model for the function of p12 in early infection (Figure 2). In the viral PIC, p12 is bound to hexameric CA via its NTD. Upon disassembly of the nuclear membranes in mitosis, the PIC is targeted and tethered to chromatin by the CTD regions of CA-bound, phosphorylated p12. Following exit from mitosis, de-phosphorylation of p12 triggers its release from chromatin. This may be concomitant with, or indeed stimulate, the dissociation of CA from the PIC, which would expose the intasome containing viral DNA and IN. By binding chromatin associated BET proteins, IN would then direct the intasome to promotor regions and catalyse the integration of the viral genome into host chromatin.

The role played by CA in the chromatin targeting of retroviruses in general and HIV-1 in particular is currently controversial. Accumulating evidence, however, indicates that different retroviral genera rely on CA-binding factors, either of viral or host origin, to target the PIC to chromatin prior to integration. In HIV-1, the host protein, CPSF6, appears to primarily fulfil this function, whereas in gammaretroviruses it is the viral protein, p12. As many orthoretroviral genera code for additional Gag-cleavage products in a similar genomic position to p12, it is tempting to speculate that they may also carry chromatin-binding sequences to direct global nuclear targeting of their viral PICs.

